# Randomized, Double-Blind, Placebo-Controlled, Dose-Ranging Comparison of the Analgesic Efficacy and Safety of Intravenous Ketoprofen, Tramadol, and Morphine After Bony Impacted Mandibular Third Molar Surgery

**DOI:** 10.7759/cureus.98017

**Published:** 2025-11-28

**Authors:** Arya Babul, Aidan Gill, Sohi Ashraf, Momina Hussain, Najib Babul

**Affiliations:** 1 Biomedical Sciences, West Career and Technical Academy, Las Vegas, USA; 2 Biomedical Sciences, Society for Awareness of Neglected Diseases, Las Vegas, USA; 3 Medical Affairs, APG-PharmaConsulting GmbH, Zug, CHE; 4 Critical Care, MountainView Hospital, Las Vegas, USA; 5 Genomics, Chinese Academy of Tropical Agriculture Sciences, Sanya, CHN; 6 Drug Development, Cinergen, LLC, Las Vegas, USA; 7 Drug Development, Quadra Therapeutics, Las Vegas, USA

**Keywords:** acute pain, ketoprofen, morphine, postsurgical pain, randomized controlled trial, seizures, third molar extraction, tramadol

## Abstract

Introduction

Although opioids remain the standard of care for managing postsurgical pain, concerns persist regarding their safety, tolerability, and potential for addiction. Intravenous (IV) formulations of ketoprofen and tramadol have been widely available in numerous countries outside the United States and Canada.

Methods

This single-dose, single-center, double-blind, randomized study (n=185) evaluated the analgesic efficacy, dose-response relationship, duration of action, and safety of IV ketoprofen 50 mg (n=33) and 100 mg (n=31), IV tramadol 100 mg (n=33) and 150 mg (n=22), IV morphine 4 mg (n=33), and placebo (n=33) administered over two minutes upon the first occurrence of moderate-to-severe pain following surgical extraction of bony impacted mandibular third molars. Pain intensity and pain relief were assessed over eight hours. The primary efficacy endpoint was total pain relief over the first four hours (TOTPAR 0-4). Secondary efficacy endpoints included TOTPAR at additional time intervals, summed pain intensity difference (SPID), peak pain relief, peak pain intensity difference, time to rescue analgesia, patient global evaluation, and time to confirmed perceptible and meaningful pain relief. Safety assessments included volunteered and observed adverse events (AEs).

Results

For TOTPAR 0-4 and all other efficacy endpoints, IV ketoprofen 50 mg and 100 mg, and IV tramadol 100 mg and 150 mg were significantly superior to IV morphine 4 mg and placebo. IV ketoprofen at both doses also outperformed IV tramadol at both doses across the majority of outcome measures. No significant differences in efficacy were observed between IV morphine 4 mg and placebo. The proportion of subjects experiencing one or more AEs was comparable among the placebo, IV ketoprofen 50 mg, and IV ketoprofen 100 mg groups. In contrast, higher AE rates were reported in the IV tramadol and IV morphine groups relative to placebo. Dosing in the IV tramadol 150 mg group was discontinued early due to the occurrence of seizures in two patients (9%) immediately following administration.

Conclusions

Both IV ketoprofen and IV tramadol demonstrated superior analgesic efficacy compared to IV morphine and placebo in the management of postsurgical pain. Notably, IV ketoprofen consistently outperformed tramadol across efficacy endpoints. The lack of efficacy observed with IV morphine 4 mg raises concerns about potential analgesic assay insensitivity in postsurgical dental pain. The occurrence of seizures in subjects without known seizure risk factors highlights a potentially insufficient safety margin for tramadol in unselected patient populations.

## Introduction

Injectable nonsteroidal anti-inflammatory drugs (NSAIDs) and opioids are widely used for postsurgical analgesia as monotherapy or within multimodal regimens aimed at accelerating recovery and minimizing adverse events (AEs). These agents are particularly suitable for patients unable to tolerate oral NSAIDs and in clinical settings necessitating IV administration, especially during the immediate postsurgical period.

Oral formulations of ketoprofen and tramadol were first approved in the US in 1986 and 1995, respectively. Tramadol and ketoprofen, in parenteral form, have been available in over 130 countries outside the U.S. since 1991 and 1997, respectively. IV tramadol is typically administered as a slow injection over two to three minutes or via infusion, whereas IV ketoprofen is commonly diluted in 100 to 150 mL of dextrose 5% in water (D5W) or normal saline and infused over 20 minutes to mitigate the risk of venous irritation [1]. Tramadol, classified as a Schedule IV controlled substance in the U.S., exerts analgesic effects through μ-opioid receptor agonism and inhibition of norepinephrine and serotonin reuptake [2]. Compared to Schedule II opioids such as fentanyl, morphine, hydromorphone, and meperidine, tramadol exhibits a lower potential for abuse.

Although the efficacy of oral ketoprofen [3-8] and oral tramadol [9-10] has been extensively evaluated in numerous double-blind, randomized, controlled trials (RCTs) in subjects with moderate to severe pain following third molar extraction, to our knowledge, no placebo-controlled studies have assessed IV ketoprofen after third molar extraction surgery. Likewise, there are no placebo-controlled RCTs comparing IV ketoprofen with IV tramadol after third molar extraction, a widely used surgical model for initial evaluation of analgesia.

This single-center, randomized, double-blind, parallel-group study evaluated the efficacy and safety of single IV doses of ketoprofen (50 mg and 100 mg) and tramadol (100 mg and 150 mg) in subjects undergoing surgical removal of ≥2 third molars, including at least one mandibular, bony impacted third molar. IV formulations of tramadol, ketoprofen, and morphine were obtained from commercially available products sourced from the United Kingdom and France. The study employed a placebo-controlled design to evaluate the efficacy, safety, and tolerability of IV ketoprofen and tramadol relative to a no-treatment condition. IV morphine 4 mg, a widely recognized standard-of-care opioid for acute postsurgical pain, was included as a positive control to assess assay sensitivity in the event of a negative outcome. To maintain treatment blinding, all test and reference analgesics were administered as a slow IV bolus over two minutes.

## Materials and methods

Ethics and registration

The study was conducted at the Eastman Dental Institute Research Center, University College Hospital, London, UK, in accordance with the Declaration of Helsinki and its amendments and the International Council for Harmonization Good Clinical Practice guidelines. It was approved by the UK Medicines and Healthcare products Regulatory Agency (MHRA). The study protocol and informed consent form were reviewed and approved by the Eastman Dental Institute and the Eastman Dental Hospital Joint Research and Ethics Committee (approval no. 03/E010) and the Quorn Research Review Committee (approval no. QRRC03103/SRX-008). Written informed consent was obtained from all participants prior to enrollment. Data for this RCT are presented in conformance with Consolidated Standards of Reporting Trials (CONSORT) guidelines.

Participants

Eligible participants were consenting male and female adults aged 18 to 40 years, in good general health, who experienced moderate or severe pain within six hours following elective surgery. Inclusion required that at least one extracted molar be a bony impacted mandibular third molar; if only two molars were removed, they were required to be ipsilateral. Female participants of childbearing potential were required to be non-lactating, have a negative pregnancy test, and be practicing abstinence or using a medically acceptable form of contraception.

Exclusion criteria included the following: presence of uncontrolled chronic disease contraindicating study participation; raised intracranial pressure, epilepsy, or history of seizure disorder (excluding juvenile febrile seizures resolved >10 years prior); recognized risk of seizure; history of asthma, urticaria, or allergic-type reactions to aspirin or NSAIDs; history of gastrointestinal bleeding, perforation, or peptic ulcer disease; other oral surgical procedures during the study; laboratory abnormalities including aspartate transaminase (AST) or alanine aminotransferase (ALT) >2× upper limit of normal, creatinine >170 µmol/L, or any finding contraindicating participation; chronic respiratory insufficiency; chronic use of analgesics or tranquilizers, or known substance abuse within the past 90 days; alcohol consumption within 12 hours prior to surgery; use of anticoagulants; use of any of the following medications within 14 days prior to dosing: monoamine oxidase inhibitors, tricyclic antidepressants or related compounds (including doxepin), neuroleptics, anorectics, bupropion, carbamazepine, quinidine, selective serotonin reuptake inhibitors (SSRIs), serotonin-norepinephrine reuptake inhibitors (SNRIs), tramadol, opioids, NSAIDs, cyclooxygenase-2 inhibitors (COX-2 inhibitors), antihistamines, tranquilizers, sedatives, hypnotics, acetaminophen, and corticosteroids.

Procedure 

Randomization

Subjects were randomly assigned to receive a single IV dose of ketoprofen 50 mg or 100 mg, tramadol 100 mg or 150 mg, morphine sulfate 4 mg, or placebo, administered over two minutes according to a computer-generated randomization schedule prepared by an independent third party. Initial randomization was conducted in a 1:1:1:1:1:1 ratio. Enrollment in the tramadol 150 mg group was discontinued after 22 subjects due to the occurrence of seizures in two participants. Subsequent randomization was adjusted to a 1:1:1:1:1 ratio among the remaining treatment arms.

Anesthetic and Surgical Protocol

During the screening visit, a clinical examination and presurgical oral radiographic imaging were performed to determine study eligibility. Subjects underwent surgical extraction of two or more third molars, provided that at least one was a bony impacted mandibular third molar; when only two third molars were extracted, they were ipsilateral. Preoperatively, patients were rinsed immediately before surgery with 10-15 mL of 0.2% chlorhexidine gluconate for 30-60 seconds as an antiseptic measure.

For mandibular extractions, an incision was typically made under local anesthesia from the anterior border of the mandibular ramus to the mesiofacial aspect of the first molar. A mucoperiosteal flap was raised, and buccal and distal bone was removed with a burr under continuous irrigation with sterile saline. Teeth were sectioned when necessary prior to removal. No other oral surgical procedures were permitted during the same session except the removal of supernumerary third molars.

All subjects received prilocaine 3% with felypressin 0.03 IU/mL. The volume of local anesthetic used was determined by the oral surgeon, typically 1-5 mL per procedure, with a maximum of 10 mL. For the mandible, inferior alveolar and lingual nerve blocks were supplemented by buccal infiltration. Where an ipsilateral third molar was removed from the maxilla, anesthesia administered at the palatal tuberosity (palatine foramen) was supplemented by buccal and palatal infiltration. Surgical trauma was classified by the surgeon as moderate (involving flap elevation and bone removal) or severe (involving flap elevation, bone removal, and tooth sectioning).

Antibiotics for prophylaxis against bacterial endocarditis or for prophylaxis or treatment of local infection were permitted.

Investigational Drug Administration

Medication assignment and administration were blinded to both participants and investigators. Study drugs were prepared in 10 mL prefilled syringes according to the randomization code immediately prior to administration by an unblinded nurse or pharmacist with no direct patient contact. The investigator administered the medication via a peripheral IV catheter. All study medications were sourced commercially: ketoprofen (Profenid IV, Sanofi-Aventis, Paris, France) as lyophilized powder for reconstitution; tramadol (Zydol, Grunenthal GmbH, Aachen, Germany) as 100 mg/2 mL vials; and morphine sulfate as 10 mg/1 mL vials (multiple sources).

Pain Assessments

Pain intensity and pain relief were assessed using self-reported scales at baseline (0 hours) and at 15, 30, and 45 minutes and at one, 1.5, two, three, four, five, six, seven, and eight hours post-dose, or immediately prior to rescue analgesia. The time points were chosen to fully characterize analgesic onset, peak effect, and duration. Pain intensity was measured using both a four-point categorical scale (None = 0, Mild = 1, Moderate = 2, Severe = 3) and a 100 mm Visual Analog Scale (VAS) anchored by “No Pain” (0 mm) and “Extreme Pain” (100 mm) in response to the statement “My pain at this time is”. Pain relief was assessed categorically on a five-point scale (None = 0, A Little = 1, Some = 2, A Lot = 3, Complete = 4) in response to the statement “My relief from starting pain is”.

Time to perceptible and meaningful pain relief was evaluated using the double stopwatch method [11]. Immediately following dosing, a trained coordinator initiated two labeled stopwatches per subject. Subjects were instructed to deactivate the first stopwatch upon experiencing perceptible pain relief and the second upon achieving meaningful pain relief. Time to confirmed perceptible pain relief was defined as the time to perceptible pain relief, only if both perceptible and meaningful pain relief were experienced.

A global evaluation of study medication was completed by each subject at eight hours or immediately prior to rescue analgesia. Subjects responded to the question, “How would you rate the study medication you received for pain?” using a five-point scale: Excellent =5, Very Good = 4, Good = 3, Fair = 2, Poor = 1. The time of evaluation was also recorded.

Subjects were encouraged to wait at least 60 minutes from the time of study medication administration before using rescue analgesia; however, rescue analgesia was permitted at any time during the study, and subjects who took rescue medication within 60 minutes after dosing were still included in the intent-to-treat (ITT) population. Pain assessments were discontinued following the first use of rescue analgesics. Immediately before the first rescue medication dose, subjects completed their final pain assessments. Rescue analgesia consisted of oral ibuprofen 400 mg or paracetamol (acetaminophen) 1 g per dose, given every four to six hours as needed (PRN), up to a maximum dose of 2.4 g of ibuprofen or 4 g of acetaminophen (paracetamol) per day. In the event that ibuprofen and/or paracetamol did not provide satisfactory analgesia, one or two doses of oral codeine 30 mg plus paracetamol 500 mg every four hours PRN could have been administered, if absolutely necessary. All study-related pain assessments were terminated following any rescue analgesic consumption.

Safety Assessments

Safety was assessed by routine laboratory analyses and physical examination performed prior to surgery and five to 10 days post-treatment. Vital signs (heart rate, respiration rate, and blood pressure) were recorded at baseline; 15 and 30 minutes; and one, four, six, and eight hours post dosing. Vital signs were collected only after pain assessments at corresponding time points. AEs were monitored during the course of the study.

Efficacy and safety outcomes 

The primary objective of this study was to compare the analgesic efficacy of IV ketoprofen (50 mg and 100 mg) and IV tramadol (100 mg and 150 mg) with placebo in subjects with moderate to severe postsurgical pain. Secondary objectives were to compare the analgesic efficacy of (i) IV ketoprofen (50 mg and 100 mg) and IV tramadol (100 mg and 150 mg) with IV morphine (4 mg); and (ii) IV ketoprofen (50 mg and 100 mg) with IV tramadol (50 mg and 100 mg); and (iii) to compare the safety of the investigational medications.

All measures of efficacy in this study were derived from the subject diary. They represent standard measures employed in single-dose analgesic studies in acute postsurgical pain models.

The primary endpoint was total pain relief (TOTPAR) over the 0-to four-hour interval. Secondary endpoints included TOTPAR over 0-to two-, 0-to six-, and 0-to eight-hour intervals; sum of pain intensity difference (SPID) over 0-to two-, 0-to four-, 0-to six-, and 0-to eight-hour intervals; time to rescue medication; time-specific pain intensity difference recorded on VAS and categorical scales; time-specific pain relief recorded on a categorical scale; peak pain intensity difference and peak pain relief; time to “pain half gone”; time to first categorical change from baseline in peak pain intensity difference of ≥ 1; time to perceptible pain relief; time to confirmed perceptible pain relief; time to meaningful pain relief; and subject global evaluation. TOTPAR and SPID scores were calculated by multiplying each time point value by the duration (in hours) since the preceding assessment and summing the resulting weighted values to generate composite scores. Safety was assessed through the incidence of AEs, changes in clinical laboratory parameters, and evaluations of vital signs and physical examination findings.

Statistical analyses

Sample Size Estimation

This preliminary study evaluated the efficacy, safety, and analgesic dose-response of intravenous ketoprofen and tramadol compared to placebo. A sample size of 30 to 50 subjects per treatment arm has consistently demonstrated treatment differences between active analgesics and placebo in single-dose studies of postsurgical dental pain, as reported in prior investigations conducted at specialized centers with highly trained research staff [12-14]. This sample size range has also served as the foundation for a majority of adequate and well-controlled single-dose efficacy trials in acute postsurgical dental pain submitted to the U.S. Food and Drug Administration (FDA). The sample size calculation for the present study was based on the hypothesis that single-dose IV ketoprofen and IV tramadol would be superior to placebo in relieving acute, moderate to severe dental pain following surgical extraction of two or more third molars, including one bony impacted mandibular molar. In the absence of prior data under these specific experimental conditions, a sample size of 30 subjects per treatment group was estimated to provide 80% power to detect superiority of IV ketoprofen over placebo for TOTPAR over the 0-four-hour interval, using a two-sided test at a 5% significance level (α = 0.05).

Handling of Dropout or Missing Data

The last observation carried forward (LOCF) method was used for early withdrawals. Efficacy assessments within five minutes of scheduled time points during the first hour and within 10 minutes after one hour were treated as on‑schedule. Missing or off‑schedule data were handled as follows: (i) for subjects who withdrew or used rescue medication before the eight‑hour assessment, the last available assessment was carried forward to subsequent time points; and (ii) missing evaluations at other time points were linearly interpolated where possible.

For total analgesic effect measures (TOTPAR, SPID), missing values at hours two, four, six, and eight were imputed as described above, using the actual time when calculating effect measures. For hourly analyses, actual times within the windows above were mapped to scheduled times, and data were imputed at each adjusted time point when missing.

For double‑stopwatch endpoints, if both stopwatches were stopped, the first and second stopwatch times were used for perceptible and meaningful relief, respectively. If only the second stopwatch had been stopped, that time would have been used for both endpoints. If only the first stopwatch had been stopped, time to meaningful relief would have been censored at eight hours. If neither stopwatch was stopped, times were censored at eight hours for subjects who took rescue medication or at the time of dropout for other discontinuations.

Efficacy and Safety Analysis

All efficacy analyses were conducted on the ITT population, defined as all randomized subjects who received study medication and had at least one post-baseline measurement of pain intensity or pain relief. To mitigate the risk of inflated Type I error due to multiplicity, multiple comparisons for each efficacy variable were performed using Fisher’s protected least significant difference (PLSD) method, which controls the comparison-wise error rate. For variables assessed across multiple time intervals, comparisons were conducted at each interval. An initial overall two-sided test (e.g., F-test or log-rank test) was evaluated for statistical significance (p ≤ 0.05); if significant, all two-sided pairwise treatment comparisons were subsequently performed, with significance declared for comparisons yielding p ≤ 0.05. TOTPAR, SPID, and time-specific pain intensity difference and pain relief were analyzed using an analysis of variance (ANOVA) model incorporating treatment and baseline pain stratification effects. Time-to-event endpoints, including time to perceptible pain relief, confirmed perceptible relief, meaningful relief, rescue medication, first pain intensity difference ≥ 1, and pain half gone, were summarized using Kaplan-Meier methodology and the Simon and Lee approach, with treatment comparisons assessed via the log-rank test within the PLSD framework. Peak pain intensity difference, peak pain relief, and subject global evaluation were analyzed using the rank-sum test, stratified by baseline pain. Categorical efficacy endpoints, including the proportion of subjects achieving 50% pain reduction, meaningful pain relief, and use of rescue analgesia, were evaluated using the chi-square test. Safety analyses were performed on the treated subject population, defined as all randomized subjects who received study medication, with outcomes summarized using descriptive statistics.

## Results

Patient disposition and demographics 

**Figure 1:**
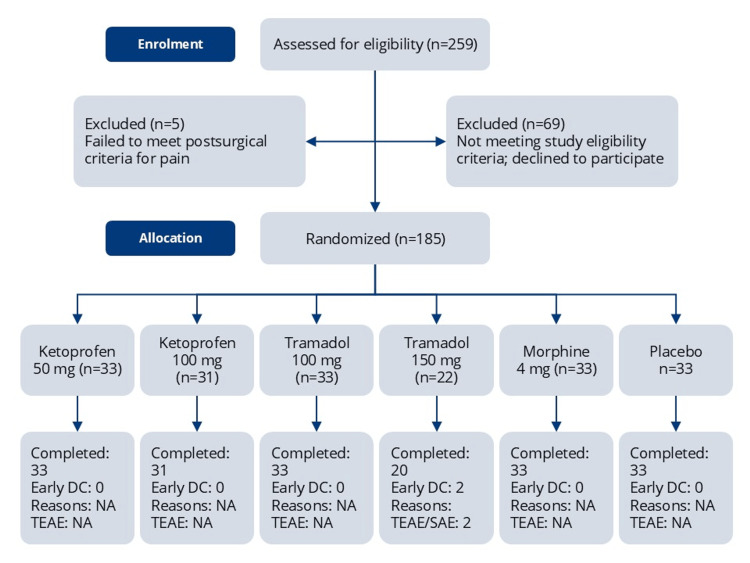
A CONSORT flow diagram of patient disposition from screening to study completion. Subjects who experienced moderate or severe pain on the categorical and Visual Analog Scale (VAS) pain intensity scales as measured by moderate or severe pain descriptor and ≥ 50 mm, respectively, and who met all other eligibility criteria were randomly assigned to receive a single dose of placebo, morphine 4 mg, tramadol 100 mg or 150 mg (terminated prematurely), or ketoprofen 50 mg or 100 mg administered IV in 10 mL prefilled syringes over two minutes. CONSORT: Consolidated Standards of Reporting Trials; DC: discontinued; NA: not applicable; TEAE: treatment-emergent adverse event; SAE: serious adverse event.

The planned target enrollment was approximately 190 subjects to ensure that 180 subjects (30 per treatment group) would complete the study. A total of 259 subjects were screened; 69 did not meet the study eligibility criteria or declined to participate, and five did not experience moderate or severe pain intensity after surgery on the categorical (moderate or severe pain descriptor) and/or the VAS (≥ 50 mm) scales (Figure 1). Subjects were initially randomized in a 1:1:1:1:1:1 ratio across six treatment groups. Enrollment in the IV tramadol 150 mg group was discontinued after 22 subjects due to the occurrence of seizures in two participants. Subsequently, randomization proceeded in a 1:1:1:1:1 ratio across the remaining five treatment groups. All 185 randomized subjects received study medication: IV ketoprofen 50 mg (n = 33) and 100 mg (n = 31), IV tramadol 100 mg (n = 33) and 150 mg (n = 22), IV morphine sulfate 4 mg (n = 33), and placebo (n = 33), and 183 were evaluable for efficacy. Demographic and baseline characteristics were generally comparable across treatment groups.

Efficacy

Ketoprofen vs. Placebo

**Figure 2:**
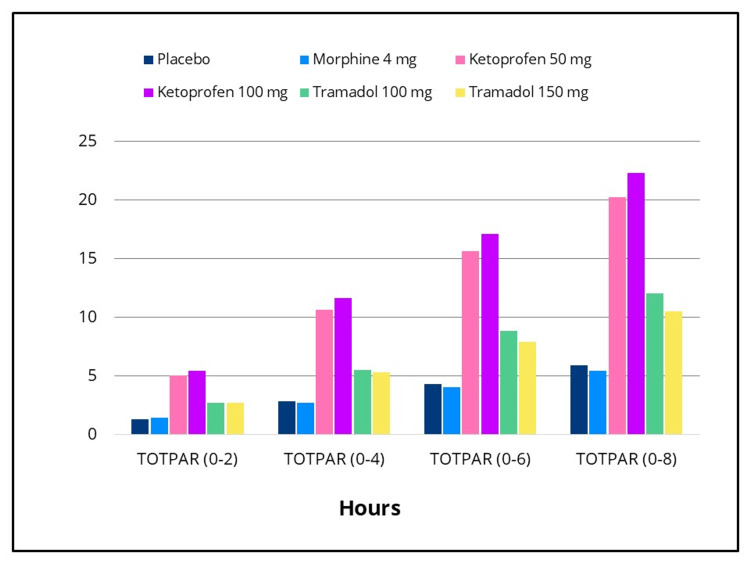
Total pain relief (TOTPAR) Over 0- to two-, 0- to four- (primary endpoint), 0- to six-, and 0- to eight-hour intervals TOTPAR over 0- to two-, 0- to four- (primary endpoint), 0- to six-, and 0- to eight-hour intervals assessed on a categorical scale in the intention-to-treat population following a single dose of placebo, morphine 4 mg, tramadol 100 mg or 150 mg (terminated prematurely), or ketoprofen 50 mg or 100 mg administered IV. Subjects responded to the question, “My relief from starting pain is” None = 0, A little = 1, Some = 2, A Lot = 3, Complete = 4 at 15, 30, and 45 minutes, and at one, 1.5, two, three, four, five, six, seven, and eight hours post dose, or at early termination. TOTPAR scores were calculated by multiplying the individual time point value by the duration (in hours) since the preceding time point and summing these weighted values.

**Figure 3:**
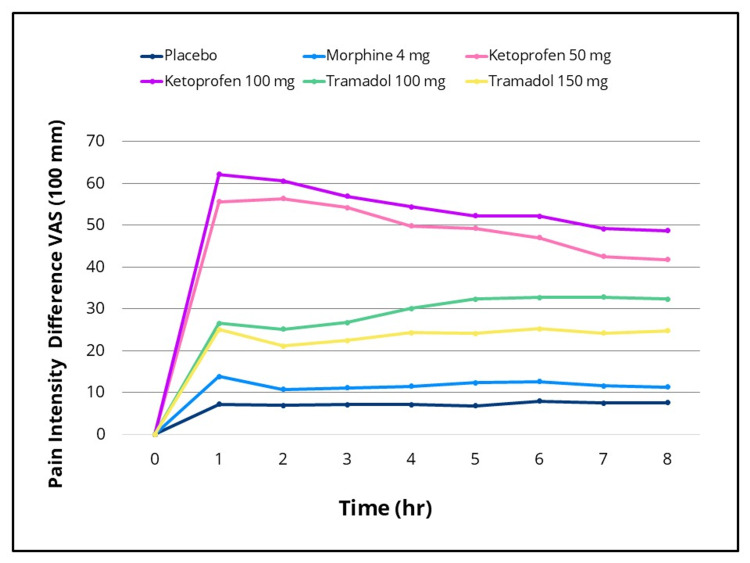
Pain intensity difference (VAS) over 0 to eight hours post dose Pain intensity difference on a 100-mm Visual Analog Scale (VAS) over the eight-hour post-dose pain assessment period in the intent-to-treat population following a single dose of placebo, morphine 4 mg, tramadol 100 mg or 150 mg (terminated prematurely), or ketoprofen 50 mg or 100 mg administered IV.  Subjects responded to the question, “My pain at this time is” by making a vertical mark on the VAS anchored by “No Pain” (0 mm) and “Extreme Pain” (100 mm) at baseline (0 hours) and at 15, 30, and 45 minutes and at one, 1.5, two, three, four, five, six, seven, and eight hours post dose, or at early termination.

**Figure 4:**
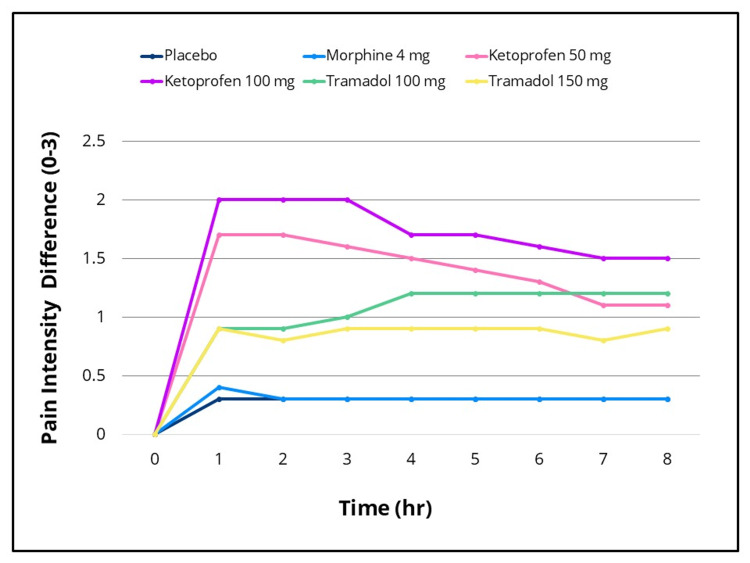
Pain intensity difference (categorical scale) over 0 to eight hours post dose Pain intensity on a four-point categorical scale in the intention-to-treat population following a single dose of placebo, morphine 4 mg, tramadol 100 mg or 150 mg (terminated prematurely), or ketoprofen 50 mg or 100 mg administered IV. Subjects responded to the question, “My pain at this time is”: None = 0, Mild = 1, Moderate = 2, Severe = 3 at baseline (0 hour), and at 15, 30, and 45 minutes, and at one, 1.5, two, three, four, five, six, seven, and eight hours post dose, or at early termination.

**Figure 5:**
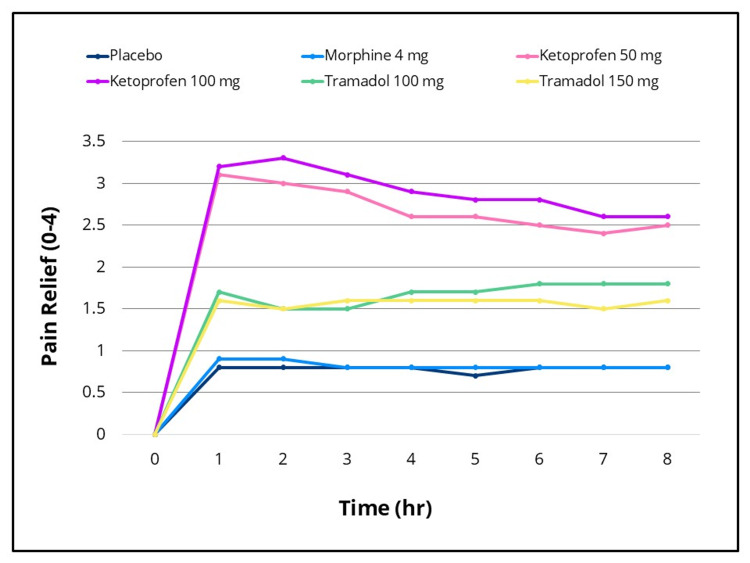
Pain relief (categorical scale) over eight hours post dose Pain relief over the eight-hour post-dose pain assessment period in the intent-to-treat population following a single dose of placebo, morphine 4 mg, tramadol 100 mg or 150 mg (terminated prematurely), or ketoprofen 50 mg or 100 mg administered IV. Subjects responded to the question, “My relief from starting pain is”: None = 0, A Little = 1, Some = 2, A Lot = 3, Complete = 4 at 15, 30, and 45 minutes, and at one, 1.5, two, three, four, five, six, seven, and eight hours post dose, or early termination.

**Figure 6:**
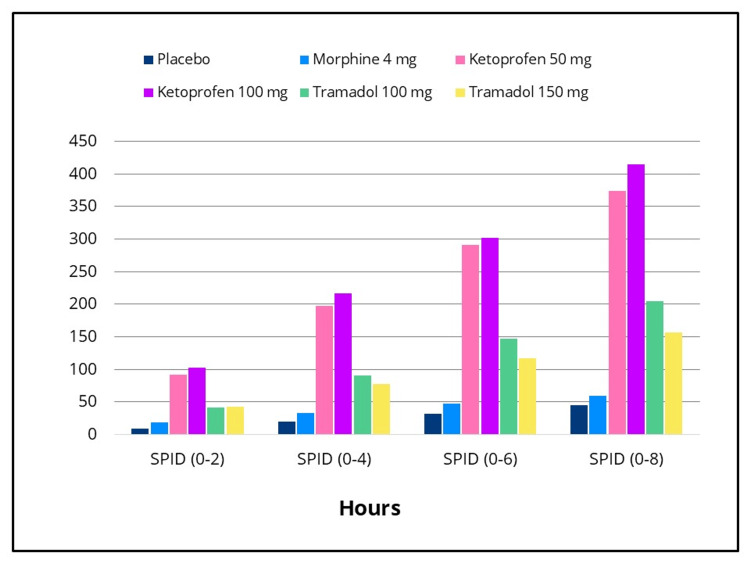
SPID (VAS) over eight hours post dose Sum of pain intensity difference (SPID) on a 100-mm Visual Analog Scale (VAS) over 0- to two-, 0- to four-, 0- to six-, and 0- to eight-hour intervals in the intent-to-treat population following a single dose of placebo, morphine 4 mg, tramadol 100 mg or 150 mg (terminated prematurely), or ketoprofen 50 mg or 100 mg administered IV. Subjects responded to the question, “My pain at this time is” by making a vertical mark on a 100 mm VAS anchored by “No Pain” (0 mm) and “Extreme Pain” (100 mm) at baseline (0 hours), and at 15, 30, and 45 minutes, and at one, 1.5, two, three, four, five, six, seven, and eight hours post-dose, or early termination. SPID scores were calculated by multiplying the individual time point value by the duration (in hours) since the preceding time point and summing these weighted values.

**Figure 7:**
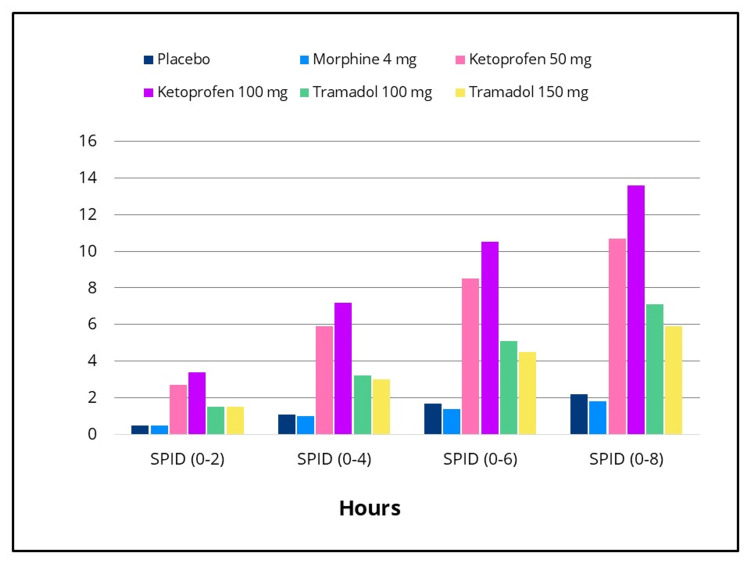
SPID (categorical scale) over eight hours post dose Sum of pain intensity difference (SPID) over the eight-hour post-dose pain assessment period in the intent-to-treat population following a single dose of placebo, morphine 4 mg, tramadol 100 mg or 150 mg (terminated prematurely), or ketoprofen 50 mg or 100 mg administered IV. Subjects responded to the question, “My pain at this time is”: None = 0, Mild = 1, Moderate = 2, Severe = 3 at baseline (0 hours), and at 15, 30, and 45 minutes, and at one, 1.5, two, three, four, five, six, seven, and eight hours post dose, or early termination. SPID scores were calculated by multiplying the individual time point value by the duration (in hours) since the preceding time point and summing these weighted values.

**Figure 8:**
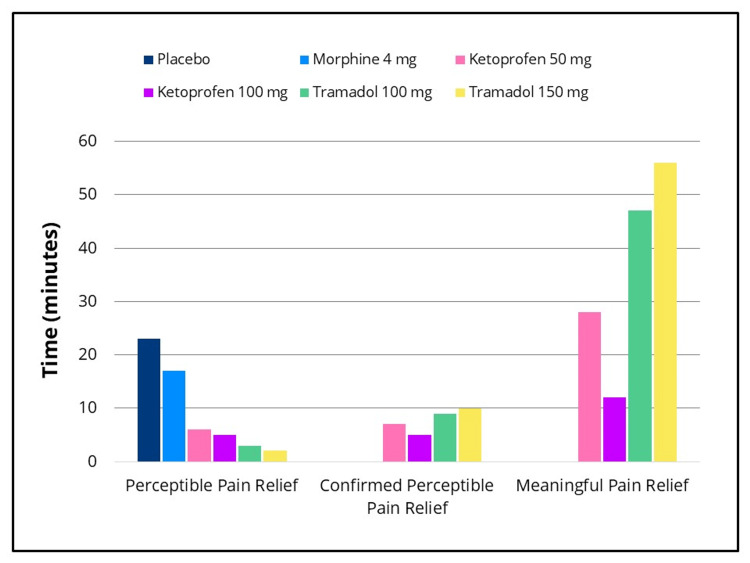
Time to perceptible and meaningful pain relief (Double Stopwatch Method) Time to perceptible pain relief (first feeling any difference in pain), confirmed perceptible and meaningful pain relief (when the relief from the pain is meaningful) measured using the double stopwatch method [11] in the intent-to-treat population following a single dose of placebo, morphine 4 mg, tramadol 100 mg or 150 mg (terminated prematurely), or ketoprofen 50 mg or 100 mg administered IV. Time to confirmed perceptible pain relief was defined as the time to perceptible pain relief, only if both perceptible and meaningful pain relief were experienced.  Note: for placebo and morphine, the median time to confirmed perceptible and meaningful relief was > 8 hours. For perceptible pain relief, subjects were instructed as follows: “I would like you to stop the first stopwatch when you first feel any pain relief whatsoever.  This does not mean you feel completely better, although you might, but when you first feel any difference in the pain that you have had.” For meaningful pain relief, subjects were instructed as follows: “I would like you to stop the second stopwatch when you have meaningful pain relief.  That is, when the relief from the pain is meaningful to you.”

**Figure 9:**
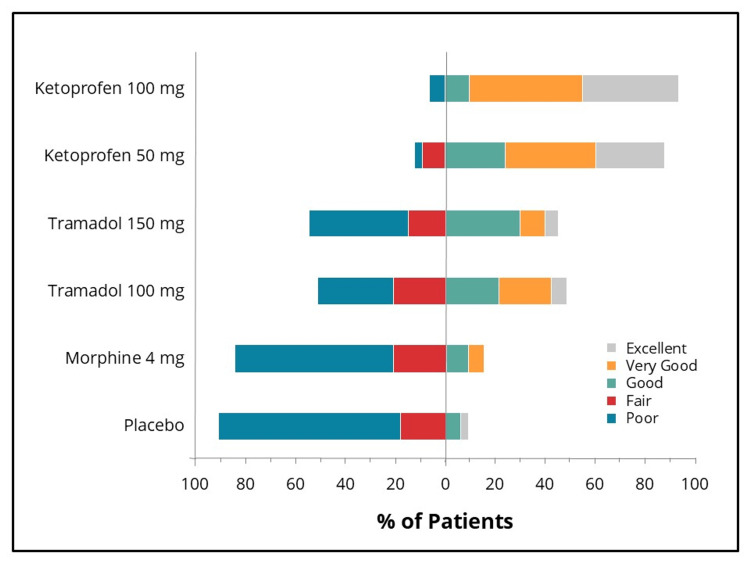
Global evaluation of study medication Global evaluation of study medication by subjects at eight hours post dose or immediately prior to rescue analgesia in the intent-to-treat population following a single dose of placebo, morphine 4 mg, tramadol 100 mg or 150 mg (terminated prematurely), or ketoprofen 50 mg or 100 mg administered IV. Subjects responded to the question, “How would you rate the study medication you received for pain?” using a five-point scale: Excellent (5), Very Good (4), Good (3), Fair (2), Poor (1), where "Good" to "Excellent" are considered favorable global evaluations.

Statistically significant differences in efficacy outcomes were observed, favoring the IV ketoprofen 50 mg and 100 mg groups over placebo for the primary endpoint, TOTPAR 0-to four- hours, and for the 0-to two-, 0-to six- and 0-to eight-hour intervals (Figure 2), as well as for all secondary efficacy measures. Pain intensity differences assessed using the 100-mm VAS and a categorical scale (0-3), along with pain relief scores on a categorical scale (0-4) over the eight-hour post-dose evaluation period, are presented in Figures 3-5, respectively. The SPID VAS and categorical values for IV ketoprofen 50 mg and 100 mg over the 0-to two-, 0-to four-, 0-to six- and 0-to eight-hour intervals are shown in Figures 6, 7, respectively. The median time to confirmed perceptible pain relief was seven minutes with IV ketoprofen 50 mg, five minutes with IV ketoprofen 100 mg, and greater than eight hours with placebo. The median time to meaningful pain relief was 28 minutes, 12 minutes, and greater than eight hours, respectively (Figure 8). The median time to rescue analgesic use was six hours and 54 minutes, more than eight hours, and one hour and 12 minutes, respectively. The proportion of patients reporting a favorable global evaluation (“Good”, “Very Good”, or “Excellent”) was 88%, 94%, and 9% for IV ketoprofen 50 mg, IV ketoprofen 100 mg, and placebo, respectively (Figure 9).

Tramadol vs. Placebo

The efficacy scores for IV tramadol 100 mg and 150 mg were also significantly superior to those of placebo across all measured outcomes. Generally, for measures of pain relief and pain intensity, mean scores for the tramadol groups were significantly better than those of the placebo for the first 45 minutes to 1.5 hours (Figures 2-7). The median time to confirmed perceptible pain relief was nine minutes with IV tramadol 100 mg and 10 minutes with IV tramadol 150 mg. The median time to meaningful pain relief was 47 minutes and 56 minutes, respectively (Figure 8). The median time to rescue medication was 2 hours and 15 minutes and 1 hour and 47 minutes, respectively (Figure 8). The proportion of patients reporting a favorable global evaluation (“Good”, “Very Good”, or “Excellent”) for IV tramadol 100 mg and 150 mg was 49% and 45%, respectively (Figure 9).

 *Ketoprofen vs. Tramadol*

The efficacy outcomes for both ketoprofen groups were significantly superior to those of either tramadol dose across nearly all measured endpoints. No statistically significant differences were observed between the ketoprofen 50 mg and 100 mg groups for most efficacy variables, with the exception of pain relief and categorical pain intensity difference at six hours, where mean scores favored the ketoprofen 100 mg group. In contrast, no statistically significant differences in efficacy outcomes were identified between the tramadol 100 mg and 150 mg groups.

Morphine

Several efficacy scores for the morphine 4 mg group were numerically, but not statistically, superior to those of the placebo group, with the exception of a single time point at which the pain relief score favored placebo. In contrast, both ketoprofen groups demonstrated superiority over morphine across all efficacy variables (Figures 2-7). Specifically, mean pain relief and pain intensity scores were significantly higher for the ketoprofen 50 mg group compared to morphine during the initial three to four hours post dose, while the ketoprofen 100 mg group showed superiority over morphine across most time points throughout the eight-hour assessment period. The tramadol groups also exhibited significant improvements over morphine for most efficacy measures, particularly during the first 45 minutes to one hour, and at various intermittent time points thereafter. In the morphine 4 mg group, the median time to confirmed perceptible pain relief and to meaningful pain relief exceeded eight hours (Figure 8). The median time to rescue medication was one hour and 38 minutes. The proportion of patients reporting a favorable global evaluation (“Good”, “Very Good”, or “Excellent”) was only 15% (Figure 9).

Summary of Pain Relief and Pain Intensity

The analgesic response in the ITT population following placebo (n = 33), IV morphine 4 mg (n = 33), tramadol 100 mg (n = 33), tramadol 150 mg (n = 20), ketoprofen 50 mg (n = 33), and ketoprofen 100 mg (n = 31), with pairwise comparisons conducted using an ANOVA model incorporating main effects for treatment and baseline pain stratification (overall treatment p-value < 0.001), is displayed in Table 1 for TOTPAR on the categorical scale (0 to two, 0 to four, 0 to six, and 0 to eight hours); Table 2 for SPID on the VAS scale (0 to two, 0 to four, 0 to six, and 0 to eight hours); and Table 3 for SPID on the categorical scale (0 to two, 0 to four, 0 to six, and 0 to eight hours). Data on other secondary efficacy variables are available upon request.

**Table 1:** Total pain relief on a categorical scale (0 to two, 0 to four, 0 to six, and 0 to eight hours) TOTPAR 0-x hours, area under curve for PR (categorical) over 0 to two, 0 to four (primary endpoint), 0 to six, and 0 to eight hours measured on a five-point scale. Subjects responded to the question: “My relief from starting pain is”: 4 = Complete, 3 = A Lot, 2 = Some, 1 = A Little, and 0 = None. ^a^Pairwise comparisons: ANOVA model was used, including main effects for treatment and baseline pain stratification in the model (overall treatment p-value was <0.001). Intent-to-treat population; ANOVA: analysis of variance; IV: intravenous; LS: least squares; PR: pain relief; Mean (SD) unless specified; TOTPAR: total pain relief

	Placebo, N = 33	Morphine 4 mg IV, N = 33	Tramadol 100 mg IV, N = 33	Tramadol 150 mg IV, N = 20	Ketoprofen 50 mg IV, N = 33	Ketoprofen 100 mg IV, N = 31
0-4 Hours (Primary)						
Mean (SD)	2.8 (3.3)	2.7 (3.3)	5.5 (4.1)	5.3 (4.7)	10.6 (3.10	11.6 (3.6)
LS Mean	2.9	2.7	5.5	5.3	10.6	11.6
P-values^a^						
vs Placebo		0.853	0.003	0.016	< 0.001	< 0.001
vs Morphine			0.002	0.011	< 0.001	< 0.001
vs Tramadol 150 mg			0.847			
vs Ketoprofen 50 mg			< 0.001	< 0.001		
vs Ketoprofen 100 mg			< 0.001	< 0.001	0.262	
0-2 Hours						
Mean (SD)	1.3 ± 1.36	1.4 ± 1.60	2.7 ± 1.84	2.7 ± 2.14	5.0 ± 1.31	5.4 ±1 .55
LS Mean	1.3	1.4	2.8	2.8	5	5.5
P-values^a^						
vs Placebo		0.877	< 0.001	0.001	< 0.001	< 0.001
vs Morphine			< 0.001	0.002	< 0.001	< 0.001
vs Tramadol 150 mg			0.938			
vs Ketoprofen 50 mg			< 0.001	< 0.001		
vs Ketoprofen 100 mg			< 0.001	< 0.001	0.215	
0-6 Hours						
Mean (SD)	4.3 ± 5.16	4.0 ± 5.13	8.8 ± 6.77	7.9 ± 7.37	15.6 ± 4.88	17.1 ± 6.00
LS Mean	4.4	4.1	8.8	8	15.6	17.1
P-values^a^						
vs Placebo		0.82	0.002	0.033	< 0.001	< 0.001
vs Morphine			0.001	0.02	< 0.001	< 0.001
vs Tramadol 150 mg			0.606			
vs Ketoprofen 50 mg			< 0.001	< 0.001		
vs Ketoprofen 100 mg			< 0.001	< 0.001	0.305	
0-8 Hours						
Mean (SD)	5.9 (7.2)	5.4 (7.0)	12.0 (9.6)	10.5 (10.0)	20.2 (6.7)	22.3 (8.5)
LS Mean	5.9	5.4	12.1	10.5	20.2	22.3
P-values^a^						
vs Placebo		0.786	0.003	0.05	< 0.001	< 0.001
vs Morphine			0.001	0.029	< 0.001	< 0.001
vs Tramadol 150 mg			0.491			
vs Ketoprofen 50 mg			< 0.001	< 0.001		

**Table 2:** SPID VAS (0 to two, 0 to four, 0 to six, and 0 to eight hours) SPID (VAS) 0-x hours was defined as the area under the curve of PID (VAS) scores over a 0-x hour interval. PI (VAS) was measured using a 100‑mm VAS. Subjects responded to the question, “My pain at this time is” by making a vertical mark on a 100 mm VAS anchored by “No Pain” (0 mm) and “Extreme Pain” (100 mm). PID at each time point was calculated as the baseline PI score minus the PI score at that time point. ^a^ Pairwise comparisons: ANOVA model was used, including main effects for treatment and baseline pain stratification in the model (overall treatment P-value was < 0.001). ANOVA: analysis of variance; IV: intravenous; LS: least squares; PI: pain intensity; PID: pain intensity difference; SD: standard deviation; SPID: sum of PID; VAS: Visual Analog Scale

	Placebo	Morphine 4 mg IV	Tramadol 100 mg IV	Tramadol 150 mg IV	Ketoprofen 50 mg IV	Ketoprofen 100 mg IV
	N = 33	N = 33	N = 33	N = 20	N = 33	N = 31
0-2 Hours						
Mean (SD)	9.1 (32.2)	18.3 (41.6)	41.7 (42.6)	41.9 (35.0)	91.6 (36.2)	102.8 (35.0)
LS Mean	8.2	18.2	40.5	40.8	91.9	102.2
P-values^a^						
vs Placebo		0.286	< 0.001	0.003	< 0.001	< 0.001
vs Morphine			0.018	0.036	< 0.001	< 0.001
vs Tramadol 150 mg			0.976			
vs Ketoprofen 50 mg			< 0.001	< 0.001		
vs Ketoprofen 100 mg			< 0.001	< 0.001	0.275	
0-4 Hours						
Mean (SD)	20.0 (78.4)	32.3 (90.4)	90.2 (102.3)	77.6 (83.4)	196.8 (82.6)	216.0 (79.5)
LS Mean	16.3	32	84.8	72.9	197.9	213.4
P-values^a^						
vs Placebo		0.468	0.002	0.023	< 0.001	< 0.001
vs Morphine			0.015	0.098	< 0.001	< 0.001
vs Tramadol 150 mg			0.626			
vs Ketoprofen 50 mg			< 0.001	< 0.001		
vs Ketoprofen 100 mg			< 0.001	< 0.001	0.476	
0-6 Hours						
Mean (SD)	32.2 (127.8)	47.3 (145.4)	146.3 (172.5)	116.8 (140.9)	291.3 (127.6)	319.0 (131.0)
LS Mean	25.5	46.6	136.5	108.2	293.3	314.1
P-values^a^						
vs Placebo		0.548	0.002	0.041	< 0.001	< 0.001
vs Morphine			0.011	0.127	< 0.001	< 0.001
vs Tramadol 150 mg			0.479			
vs Ketoprofen 50 mg			< 0.001	< 0.001		
vs Ketoprofen 100 mg			< 0.001	< 0.001	0.556	
0-8 Hours						
Mean (SD)	45.4 (179.1)	59.5 (199.8)	204.0 (245.8)	156.3 (200.6)	373.2 (172.9)	414.3 (183.9)
LS Mean	35.6	58.6	189.7	143.7	376.1	407.2
P-values^a^						
vs Placebo		0.64	0.002	0.056	< 0.001	< 0.001
vs Morphine			0.008	0.13	< 0.001	< 0.001
vs Tramadol 150 mg			0.411			
vs Ketoprofen 50 mg			< 0.001	< 0.001		
vs Ketoprofen 100 mg			< 0.001	< 0.001	0.53	

**Table 3:** SPID, categorical scale (0 to two, 0 to four, 0 to six, and 0 to eight hours) SPID (categorical) 0-x hours was defined as the area under the curve of PID (categorical) scores over a 0-x hour interval. PI (categorical) was measured using a 4‑point scale. Subjects responded to the question, “My pain at this time is”: None = 0, Mild = 1, Moderate = 2, Severe = 3.  PID at each time point was calculated as the baseline PI score minus the PI score at that time point. ^a^ Pairwise comparisons: ANOVA model was used, including main effects for treatment and baseline pain stratification in the model (overall treatment p-value was < 0.001). ANOVA: analysis of variance; IV: intravenous; LS: least squares; PI: pain intensity; PID: pain intensity difference; SD: standard deviation; SPID: sum of PID

	Placebo	Morphine 4 mg IV	Tramadol 100 mg IV	Tramadol 150 mg IV	Ketoprofen 50 mg IV	Ketoprofen 100 mg IV
	N = 33	N = 33	N = 33	N = 20	N = 33	N = 31
0-2 Hours						
Mean (SD)	0.5 ± 1.12	0.5 ± 1.30	1.5 ± 1.39	1.5 ± 1.47	2.7 ± 1.14	3.4 ± 1.42
LS Mean	0.3	0.5	1.2	1.3	2.8	3.2
P-values^a^						
vs Placebo		0.422	< 0.001	0.002	< 0.001	< 0.001
vs Morphine			0.012	0.019	< 0.001	< 0.001
vs Tramadol 150 mg			0.873			
vs Ketoprofen 50 mg			< 0.001	< 0.001		
vs Ketoprofen 100 mg			< 0.001	< 0.001	0.14	
0-4 Hours						
Mean (SD)	1.1 ± 2.43	1.0 ± 3.01	3.2 ± 3.31	3.0 ± 3.39	5.9 ± 2.52	7.2 ± 3.34
LS Mean	0.6	0.9	2.5	2.4	6	6.9
P-values^a^						
vs Placebo		0.603	0.003	0.016	< 0.001	< 0.001
vs Morphine			0.015	0.052	< 0.001	< 0.001
vs Tramadol 150 mg			0.859			
vs Ketoprofen 50 mg			< 0.001	< 0.001		
vs Ketoprofen 100 mg			< 0.001	< 0.001	0.183	
0-6 Hours						
Mean (SD)	1.7 ± 3.71	1.4 ± 4.88	5.1 ± 5.56	4.5 ± 5.48	8.5 ± 3.94	10.5 ± 5.46
LS Mean	0.9	1.4	4.1	3.5	8.8	10
P-values^a^						
vs Placebo		0.642	0.003	0.029	< 0.001	< 0.001
vs Morphine			0.012	0.076	< 0.001	< 0.001
vs Tramadol 150 mg			0.666			
vs Ketoprofen 50 mg			< 0.001	< 0.001		
vs Ketoprofen 100 mg			< 0.001	< 0.001	0.246	
0-8 Hours						
Mean (SD)	2.2 ± 5.02	1.8 ± 6.72	7.1 ± 7.92	5.9 ± 7.60	10.7 ± 5.43	13.6 ± 7.62
LS Mean	1.1	1.7	5.6	4.6	11	12.8
P-values^a^						
vs Placebo		0.693	0.002	0.04	< 0.001	< 0.001
vs Morphine			0.009	0.088	< 0.001	< 0.001
vs Tramadol 150 mg			0.544			
vs Ketoprofen 50 mg			< 0.001	< 0.001		
vs Ketoprofen 100 mg			< 0.001	< 0.001	0.236	

Safety

The proportion of subjects reporting at least one AE was comparable across the placebo (54.5%), ketoprofen 50 mg (60.6%), and ketoprofen 100 mg (64.5%) groups. In contrast, AE rates were approximately 18% to 32% higher in the morphine 4 mg (72.7%), tramadol 100 mg (72.7%), and tramadol 150 mg (86.4%) groups relative to placebo. The most frequently reported AEs in the ketoprofen groups included headache, hypoesthesia (localized pain near the IV site), oral hypoesthesia, application site burning, somnolence, application site swelling, injection site irritation, postoperative infection, and contusion. A dose-dependent increase in the incidence of hypoesthesia, application site swelling, injection site irritation, and postoperative infection was observed, while somnolence occurred exclusively in the ketoprofen 100 mg group. The majority of AEs in the ketoprofen groups were mild or moderate in intensity and considered related to study medication. Headache and hypoesthesia were the most commonly reported related AEs, each occurring in five subjects across the two ketoprofen groups. 

In the tramadol groups, the most frequently reported AEs included dizziness, nausea, vomiting, application site swelling, postsurgical infection, somnolence, headache, contusion, application site ulceration, seizures, postoperative hematoma, ear pain, and blurred vision. The incidence of most AEs appeared to increase with escalating dose. While the majority of AEs in the tramadol 100 mg group were mild or moderate in intensity, all AEs in the tramadol 150 mg group were classified as moderate or severe. Nausea, dizziness, and vomiting were the most commonly reported severe AEs. Most AEs in the tramadol groups were considered related to the study medication. The most frequently reported related AEs across both tramadol groups were dizziness (23 subjects), nausea (18 subjects), and vomiting (11 subjects).

In the morphine 4 mg group, the most frequently reported AEs were dry mouth, vomiting, application site swelling, nausea, ear pain, and flushing. Severe AEs were observed in three of 33 subjects. The majority of AEs were considered related to the study medication, with vomiting and nausea being the most commonly reported related events.

Serious AEs (SAEs) were reported in three subjects receiving tramadol 150 mg. Two subjects experienced seizures immediately following administration, which were assessed by the investigator as probably related to the study medication; both subjects were subsequently discontinued from the study. The third subject was hospitalized for a postsurgical abscess, deemed unrelated to the study drug. Due to the seizure-related SAEs, enrollment in the tramadol 150 mg group was halted. No SAEs or study discontinuations occurred in any other treatment group. No clinically significant changes in laboratory parameters or vital signs were observed across treatment groups, with the exception of one subject in the morphine 4 mg group who experienced severe hypotension accompanied by loss of consciousness.

## Discussion

In this single-dose, single-center, double-blind, randomized study, we evaluated the analgesic efficacy and safety of IV ketoprofen (50 mg and 100 mg), IV tramadol (100 mg and 150 mg), IV morphine (4 mg), and placebo, each administered over a two-minute infusion in 185 subjects experiencing moderate to severe postsurgical pain. For the primary endpoint, TOTPAR 0-4, both doses of IV ketoprofen and IV tramadol demonstrated superior efficacy compared to IV morphine and placebo. Across all secondary efficacy variables, IV ketoprofen and IV tramadol were significantly more effective than IV morphine and placebo. Furthermore, IV ketoprofen (50 mg and 100 mg) consistently outperformed IV tramadol (100 mg and 150 mg) across the majority of efficacy measures.

Injectable formulations of ketoprofen and tramadol have been available in numerous countries outside the US and Canada for over four decades. IV tramadol is typically administered as a slow bolus over two to three minutes or via infusion, whereas IV ketoprofen is commonly delivered as a 20-minute infusion. For the purposes of blinding, all study medications were standardized to a two-minute administration. This approach yields a higher peak plasma concentration (Cmax), which has the potential to enhance the onset of analgesia. For instance, in a study comparing IV ketoprofen bolus with 90- and 120-minute infusions in patients with renal colic, bolus administration resulted in a more rapid onset of analgesia [1]. However, for agents with narrower safety margins, such as tramadol, elevated Cmax levels may increase the risk of AEs associated with transiently higher peak concentrations.

Although IV tramadol 150 mg has been evaluated in prior studies involving healthy volunteers [15] and subjects with postsurgical pain [16-18], current prescribing guidance recommends an initial 100 mg bolus, followed by 50 mg increments every 10 to 20 minutes as needed within the first hour, up to 250 mg. Maintenance dosing thereafter consists of 50-100 mg every four to six hours, not to exceed 400 mg (600 mg at the time of the study) daily [19-20]. In this study, two subjects, a 23-year-old female and a 30-year-old male, experienced seizures within four minutes of receiving IV tramadol 150 mg. The seizures, deemed life-threatening by the investigator, led to Research Review Committee consultation, manufacturer notification, and immediate termination of the IV tramadol 150 mg arm. Both subjects recovered following supportive care. Notably, the events occurred despite the exclusion of individuals with known seizure risks or predisposing factors.

Dosing with IV morphine was limited to 4 mg over a two-minute period, based on concerns that a higher and more rapidly infused dose could precipitate acute opioid toxicity, manifesting as respiratory depression and sedation, and potentially also confound accurate pain assessments. However, IV morphine at 4 mg failed to yield clinically significant analgesia in subjects experiencing moderate to severe pain following surgical extraction of impacted third molars. Although this dose modestly exceeds the typical 0.5-3 mg per activation range of patient-controlled analgesia regimens for postsurgical pain, the lack of efficacy in our study may reflect both limitations of the dental pain model in detecting opioid responsiveness and the absence of a loading dose or a continuous background infusion. Christensen [12] demonstrated efficacy for IV morphine 7.5 mg compared to placebo in postsurgical dental pain. However, their methodology differed substantially: (i) extractions involved ≥3 third molars, including ≥2 bony impacted mandibular molars; (ii) procedures were performed under general anesthesia; and (iii) IV morphine was infused over 10 minutes, potentially enhancing the therapeutic index and safety profile. In contrast, Minkowitz [21] recently demonstrated efficacy for both IV tramadol 50 mg and IV morphine 4 mg versus placebo in postsurgical pain following abdominoplasty. These findings suggest that opioid monotherapy may exhibit limited assay sensitivity in the postsurgical dental impaction pain model.

Analgesic clinical trials conducted following the surgical removal of impacted third molars offer a highly controlled and methodologically rigorous framework for evaluating the efficacy and safety of single-dose analgesics. The contemporary version of this dental impaction model was originally developed by Cooper and subsequently optimized by Cooper, Mehlisch, Desjardins, Dionne, Hargreaves, Hersh, Sunshine, and other investigators. It has since been employed in hundreds of analgesic clinical trials [22]. As highlighted by Singla, Desjardins, and Chang, the model benefits from the elective nature of the procedure, the use of investigational agent monotherapy, localized and uniform surgical trauma, minimal comorbidity burden, and a predominantly young and healthy study population. Furthermore, subject enrollment in this setting is substantially faster than in other postsurgical pain models, such as bunionectomy, joint arthroplasty, or soft tissue surgery [23, 24].

Despite its widespread use, several limitations of the postsurgical dental pain model have been cited by clinicians and regulators, including presumed lower pain intensity, limited generalizability to the broader postsurgical pain population, reduced assay sensitivity for opioid monotherapy, and the relatively short duration of pain [23]. However, some of these concerns are not substantiated by data from randomized controlled trials. For instance, the standardized effect size observed in the dental pain model significantly exceeds that reported in other postsurgical pain models, including bunionectomy, joint replacement, and soft tissue surgery, likely attributable to the use of highly trained study personnel operating within one or a few dedicated research centers under tightly controlled conditions [23, 24]. 

Nonetheless, there remains a strong rationale for evaluating investigational analgesics in postsurgical soft-tissue pain and in study populations that allow for repeated dosing over multiple days, including older adults with comorbidities, to assess single-entity opioids and recruit more diverse and clinically representative patient cohorts. For example, abdominoplasty, an elective cosmetic surgical procedure involving the removal of excess skin and adipose tissue and the tightening of abdominal muscles and fascia, has more recently been pioneered by Singla as a well-validated alternative surgical model for analgesic evaluation [24]. Although it requires a larger sample size and involves more invasive surgery, abdominoplasty offers many of the same advantages as the dental impaction model and appears to have greater clinical and regulatory receptivity.

In the present study, the overall incidence of AEs following a single IV dose of ketoprofen at either 50 mg or 100 mg was comparable to that observed with placebo and markedly lower than that reported for tramadol and morphine. Most AEs in the ketoprofen cohorts were of mild to moderate intensity. In patients experiencing moderate to severe pain following surgical extraction of bony impacted mandibular third molars, IV ketoprofen demonstrated favorable tolerability and elicited a robust analgesic response, outperforming tramadol, morphine, and placebo. Although IV tramadol at 100 mg exhibited analgesic efficacy, its response was significantly inferior to that of ketoprofen and was associated with a higher incidence of AEs. Notably, the occurrence of seizures in 9% of participants receiving IV tramadol at 150 mg, despite rigorous screening for seizure risk factors, raises serious safety concerns [23]. These findings suggest that even a single bolus dose of tramadol at 100 mg may provide an insufficient safety margin and pose unacceptable risks in real-world clinical settings. Accordingly, we recommend that IV tramadol for postsurgical pain be administered either as a low-dose repeated bolus (e.g., 25-50 mg) or via controlled infusion when doses exceed 50 mg [25].

## Conclusions

This single-dose, single-center, double-blind, randomized study evaluated the analgesic efficacy, dose-response relationship, duration of action, and safety of IV ketoprofen (50 mg and 100 mg), IV tramadol (100 mg and 150 mg), IV morphine (4 mg), and placebo in subjects experiencing moderate-to-severe pain following surgical removal of ≥2 third molars, including at least one mandibular, bony impacted molar.

Both IV ketoprofen and IV tramadol demonstrated superior analgesic efficacy compared to IV morphine and placebo. IV ketoprofen consistently outperformed tramadol; the analgesic effect of IV tramadol (100 mg and 150 mg) was approximately half that of IV ketoprofen at both doses. The limited efficacy of IV morphine 4 mg may reflect analgesic assay insensitivity in postsurgical dental pain. Notably, seizures occurred in two subjects (9%) receiving IV tramadol 150 mg despite no known seizure risk, suggesting a potentially insufficient safety margin in unselected patient populations.
